# Time of the day and season distribution among stroke code subtypes: differences between ischemic stroke, intracranial hemorrhage, and stroke mimic

**DOI:** 10.3389/fneur.2024.1372324

**Published:** 2024-03-26

**Authors:** Alex Menéndez Albarracín, Adrián Valls Carbó, Neus Rabaneda Lombarte, Bárbara Yugueros Baena, Jaime Carbonell Gisbert, Belén Flores-Pina, Maria-Clara Larrañaga De Bofarull, Marina Martínez Sánchez, María Hernández-Pérez, Alejandro Bustamante Rangel, Laura Dorado Bouix, Meritxell Gomis Cortina, Mònica Millán Tornè, Natalia Pérez de la Ossa

**Affiliations:** Hospital Germans Trias i Pujol, Badalona, Spain

**Keywords:** stroke, chronobiological rhythm, circadian rhythm, stroke code, haemorrhagic stroke

## Abstract

**Background:**

Circadian variations in the timing of the onset of stroke symptoms have been described, showing a morning excess of cardiovascular risk. To date, no differences have been found between stroke subtype and time distribution throughout the day. The present study aims to compare the seasonal and circadian rhythm of symptoms onset in ischemic, hemorrhagic, and stroke mimic patients.

**Methods:**

This study was conducted prospectively at a hospital and involved a cohort of stroke alert patients from 2018 to 2021. Stroke subtypes were classified as ischemic stroke, intracerebral hemorrhage (ICH), transient ischemic attack (TIA), and stroke mimic. Clinical variables were recorded, and each patient was assigned to a 4-h interval of the day according to the time of onset of symptoms; unwitnessed stroke patients were analyzed separately. Seasonal changes in stroke distribution were analyzed at 3-month intervals.

**Results:**

A total of 2,348 patients were included in this analysis (ischemic 67%, ICH 13%, mimic 16%, and TIA 3%). Regardless of stroke subtype, most of the patients were distributed between 08–12 h and 12–16 h. Significant differences were found in the time distribution depending on stroke subtype, with ICH predominating in the 4–8 h period (dawn), most of which were hypertensive, TIA in the 12–16 h period (afternoon), and stroke mimic in the 20 h period (evening). The ischemic stroke was evenly distributed throughout the different periods of the day. There were no differences in the seasonal pattern between different stroke subtypes, with winter being the one that accumulated the most cases.

**Conclusion:**

The present study showed different circadian patterns of stroke subtypes, with a predominance of ICH at dawn and stroke mimic in the afternoon. The stroke circadian rhythm resembles previous studies, with a higher incidence in the morning and a second peak in the afternoon.

## Background and purpose

The influence of circadian rhythm on cardiovascular diseases, and in particular, on the distribution of stroke incidence throughout the day, has been previously addressed (1–4). Intrinsic changes in the individual, such as vascular tone, blood pressure (BP), coagulation balance, and medication effects, vary depending on the time of day and thus create a specific time distribution to the onset of symptoms (5, 6).

Previous studies reveal a bimodal distribution, with a peak-level incidence of ischemic stroke in the morning hours and a second, less pronounced peak in the afternoon (7, 8). Ischemic and intracerebral hemorrhage (ICH) seem to have a similar circadian distribution (2, 3, 8, 9). Several underlying mechanisms related to the circadian variation of stroke onset have been described that may explain the predominance in the hours around awakening, such as disregulations in BP, especially in reverse-dippers (10, 11), a higher incidence of atrial fibrillation (12, 13), and elevated levels of cortisol, blood viscosity, and platelet aggregability related to the transition to an upright posture (14–19).

Regarding seasonal trends, previous studies have found an association between ischaemic events and seasonal changes throughout the year, with conflicting results. Some studies have found a positive association with colder months (20–22), while others have found no association (23) or a peak incidence in warmer months (24). The mortality rate seems to be unquestionably higher during colder months (25, 26).

Many unanswered questions remain about chronobiological patterns in different stroke subtypes. On the other hand, previous studies have not focused on patients with stroke code activation, so they do not include mimic stroke as a diagnostic category, which includes other diagnostics such as syncope, seizures, migraine auras, infectious disease, hypoglycemia, and functional neurological disorders (27). These data might be useful in the pre-hospital and hospital care of patients, as it is being incorporated in artificial intelligence-based predictive models of stroke subtypes or reperfusion candidates. The objective of this study is to describe the characteristics and circadian variation in the symptom onset according to the final diagnosis of stroke code alerts, as well as the temporal profile throughout the day regarding the severity of the symptoms.

## Materials and methods

### Study design

A prospective, hospital-based study was conducted on a cohort of patients admitted to the Emergency Room of a Comprehensive Stroke Center covering an area of approximately 800.000 inhabitants with a suspicion of an acute stroke between January 2018 and December 2021. All stroke suspicions are included in a prospective registry integrated into the electronic medical record (*procés ictus*) mandatory by the Public Health Catalan Department. This registry is filled out by the attending neurologists during hospitalization.

### Patients and variables

Inclusion criteria were patients with a suspicion of stroke in which the stroke code protocol was activated.

In Catalonia, a territorial stroke code protocol is standardized with the following criteria: (1) acute onset of neurological focal symptoms, (2) symptoms onset within 8 h or unknown onset, (3) patients older than 18 years old, and (4) previously functionally independent for everyday life activities. A stroke code is activated by the Emergency Medical Service (EMS) or the emergency department of any hospital upon suspicion of a stroke case, and patients are then transferred by EMS to the nearest stroke center within a network of 29 stroke centers. EMS professionals attend to the patient, record vital constants, evaluate the clinical severity using the Rapid Arterial oCclusion Evaluation Scale (RACE) scale (28), and pre-notify the receiving stroke center before the arrival of the patient.

Patients who arrived without stroke code activation were not included because the precise time of symptom onset was difficult to establish in most of them. Additionally, patients transferred from other stroke centers already diagnosed with ischemic stroke and candidates for endovascular treatment, in-hospital stroke patients already admitted to the hospital for other causes, and patients with insufficient data were excluded from the analysis. Spinal stroke, subarachnoid hemorrhage, and venous sinus thrombosis were also excluded from the final analysis.

The diagnosis of stroke was defined according to the updated World Health Organization criteria as a rapidly developed clinical sign of focal (or global) disturbance of cerebral function lasting more than 24 h or leading to death, with no apparent cause other than vascular origin. It was further classified as ischemic stroke, ICH, transient ischemic attack (TIA) (no neuroimaging damage and full recovery in the first 24 h), and stroke mimic (stroke symptoms caused by another identifiable cause) (29, 30).

Demographic data and medical history were recorded for each patient, including age, sex, smoking and alcohol habits, hypertension, dyslipidemia, diabetes mellitus, ischemic heart disease, atrial fibrillation, peripheral arteriopathy, and previous history of stroke. On arrival, vital signs and clinical variables were recorded: temperature, systolic and diastolic BP, glycemia, pre-hospital RACE scale, National Institute of Health Stroke Scale (NIHSS), laterality of stroke, the origin of stroke code alert, and hospital arrival time. After clinical evaluation, a neuroimaging (CT or MR) and vascular assessment were performed to obtain the final diagnosis of ischemic stroke, ICH, TIA, or stroke mimic. ICH was further categorized by its etiology as hypertensive versus other causes, which include vascular malformations, amyloid angiopathy, and unknown etiology.

For the purpose of the study, each patient was assigned to a 4-h interval slot of the day depending on when the symptoms started: 0–4 h (night), 4–8 h (dawn), 8–12 h (morning), 12–16 h (afternoon), 16–20 h (early evening), and 20 h (evening). Patients with unknown symptoms onset, including wake-up strokes, were not classified in any time slot and instead were studied as a separate group with no time slot division.

Stroke distribution throughout the year was also studied. Each stroke subtype was classified by trimester: winter (January–March), spring (April–June), summer (July–September), and fall (October–December).

### Statistical analysis

Baseline characteristics were compared between patients in each stroke subtype category using Fisher’s exact, the chi-square, or the Kruskal–Wallis rank sum test as appropriate. Pairwise comparison tests were performed when the global test was significant, using Fisher’s exact, the chi-square, or Dunn’s test when appropriate.

The analysis of the temporal distribution of cases in each group was performed using the chi-square test. To detect which cells are more associated with temporal distribution, we performed a description of standardized residuals. Standardized residuals are a function of the observed and expected values in each cell of the chi-square test according to the formula. Big positive residuals mean an excess of cases (positive association), while big negative residuals mean a lack of cases (negative association). The standardized residuals of the chi-square test (the difference between observed and expected values) for the temporal distribution of cases by stroke subtype are shown graphically.

We considered the number of cases per season as a time series over the course of the study. Using seasonal-trend decomposition with LOESS (STL), we obtained trend, seasonality, and remainder components from the series. Trend and seasonality strengths were evaluated, and the presence of seasonality was checked using a two-sided Kruskal–Wallis test (31, 32).

For the temporal variations of NIHSS and arrival time, a Kruskal–Wallis test was performed to test the difference between time intervals. When the test was significant, a *post-hoc* pairwise test (Dunn’s test) was performed.

Numerical values were expressed by median and interquartile range [IQR], while categorical values were expressed by number and percentage.

Two-sided values with a *p*-value of ≤0.05 were considered statistically significant, and when required, the *p*-value was adjusted using the Benjamini-Hochberg method. Statistical analyses and graphical representations were performed using R and RStudio.

### Ethics

This is a real-world evidence analysis using a local stroke registry, which satisfies all legal requirements mandated by the local law of personal data protection. Participants admitted to the Stroke Unit fulfilled informed consent for the use of clinical and radiological data, and written informed consent was obtained for participation in this study. The Ethical Committee at Germans Trias i Pujol University Hospital approved the study (registry code PI-19-080). The dataset was processed and analyzed according to local and European laws: Regulation (EU) 2016/679 of the European Parliament and of the Council of April 27, 2016, on Data Protection and Spanish Organic Law 3/2018, of 5 December, on Protection of Personal Data and Guarantee of Digital Rights.

## Results

From a total of 4,919 patients who attended our hospital, we excluded 1,744 patients (35.4%) who were admitted with no stroke code alert, 223 (4.5%) referred from other stroke centers for endovascular treatment, 272 (5.5%) in-hospital stroke alert already admitted in the hospital for other pathologies, 40 (0.7%) suffering other vascular neurological syndromes (spinal stroke, subarachnoid hemorrhage, and venous sinus thrombosis), and 292 (5.9%) with missing data on the time of symptoms onset or arrival at the hospital. Finally, 2,348 patients with stroke alertness were included in this analysis.

Baseline characteristics according to the stroke subtype are shown in Table 1. The frequency of ischemic stroke was the highest (67%), followed by stroke mimic (16%), ICH (13%), and TIA (3%).

**Table 1:** Baseline characteristics, risk factors, and stroke characteristics on hospital arrival.

	[ALL] *N* = 2,348	Ischemic stroke *N* = 1,589	Hemorrhagic stroke *N* = 300	TIA *N* = 89	Mimic *N* = 370	*p*-value
**Demographic and risk factors**						
Age	73.0 [61.5;81.0]	73.0 [63.0;82.0]	73.5 [64.0;81.0]	72.0 [59.0;78.0]	70.0 [56.0;78.8]	<0.001
Sex						0.053
Female (*n*, %)	989 (42.1%)	672 (42.3%)	109 (36.3%)	35 (39.3%)	173 (46.8%)	
Male (*n*, %)	1,359 (57.9%)	917 (57.7%)	191 (63.7%)	54 (60.7%)	197 (53.2%)	
Hypertension (*n*, %)	1,265 (53.9%)	1,012 (63.7%)	196 (65.3%)	43 (48.3%)	14 (3.78%)	<0.001
Diabetes mellitus (*n*, %)	401 (17.1%)	336 (21.1%)	43 (14.3%)	14 (15.7%)	8 (2.16%)	<0.001
Dyslipidemia (*n*, %)	980 (41.7%)	811 (51.0%)	119 (39.7%)	38 (42.7%)	12 (3.24%)	<0.001
Obesity (*n*, %)	253 (10.8%)	210 (13.2%)	33 (11.0%)	9 (10.1%)	1 (0.27%)	<0.001
Smoking habit (*n*, %)	147 (9.04%)	116 (8.83%)	23 (9.66%)	8 (12.5%)	0 (0.00%)	
Alcoholic habit (*n*, %)	154 (6.56%)	121 (7.61%)	27 (9.00%)	4 (4.49%)	2 (0.54%)	<0.001
Peripheral arthery disease (*n*, %)	134 (5.71%)	117 (7.36%)	11 (3.67%)	5 (5.62%)	1 (0.27%)	<0.001
Atrial fibrilation (*n*, %)	388 (16.5%)	316 (19.9%)	56 (18.7%)	13 (14.6%)	3 (0.81%)	<0.001
History of stroke (*n*, %)	269 (11.5%)	226 (14.2%)	29 (9.67%)	9 (10.1%)	5 (1.35%)	<0.001
Anticoagulation (*n*, %)	321 (13.7%)	241 (15.2%)	64 (21.3%)	12 (13.5%)	4 (1.08%)	<0.001
**Stroke characteristics**						
Wake up stroke/uncertain onset (*n*,%)	711 (30.3%)	581 (36.6%)	106 (35.3%)	12 (13.5%)	12 (3.2%)	<0.001
Onset to ED arrival time, (min), median [IQR]	170 [79.0;509]	179 [80.0;553]	146 [76.2;385]	118 [66.8;235]	157 [80.0;373]	0.026
Systolic BP (mmHg), median [IQR]	140 [123;160]	139 [123;159]	144 [126;168]	142 [125;166]	144 [126;170]	0.030
Dyastolic BP (mmHg), median [IQR]	77.0 [68.0;87.0]	77.0 [68.0;87.0]	79.0 [69.0;90.0]	78.0 [70.0;89.0]	85.0 [74.2;104]	0.010
Baseline NIHSS, median [IQR]	7.00 [3.00;17.0]	6.00 [3.00;15.0]	17.0 [9.00;23.0]	0.00 [0.00;1.00]	7.00 [3.00;10.0]	<0.001
RACE scale, median [IQR]	5.00 [2.00;7.00]	4.00 [2.00;7.00]	6.00 [4.00;7.00]	2.00 [1.00;4.00]	3.00 [3.00;6.00]	<0.001
Laterality						
Bilateral (*n*, %)	10 (0.53%)	6 (0.40%)	4 (1.43%)	0 (0.00%)	0 (0.00%)	0.267
Right (*n*, %)	720 (38.5%)	565 (37.7%)	124 (44.3%)	24 (32.0%)	7 (41.2%)	
Left (*n*, %)	908 (48.5%)	745 (49.7%)	115 (41.1%)	41 (54.7%)	7 (41.2%)	
Vertebrobasilar (*n*, %)	117 (6.25%)	89 (5.94%)	21 (7.50%)	5 (6.67%)	2 (11.8%)	
Indeterminate (*n*, %)	60 (3.21%)	43 (2.87%)	12 (4.29%)	4 (5.33%)	1 (5.88%)	
Oxford classification (*n*, %)						<0.001
LACI (*n*, %)	350 (19.1%)	301 (20.0%)	29 (11.6%)	20 (31.2%)	0 (0.00%)	
PACI (*n*, %)	623 (34.1%)	537 (35.7%)	46 (18.3%)	36 (56.2%)	4 (40.0%)	
POCI (*n*, %)	239 (13.1%)	201 (13.4%)	29 (11.6%)	7 (10.9%)	2 (20.0%)	
TACI (*n*, %)	617 (33.7%)	465 (30.9%)	147 (58.6%)	1 (1.56%)	4 (40.0%)	
Source of SC alert						
EMS (*n*, %)	1,083 (56.8%)	841 (55.2%)	204 (70.8%)	28 (36.4%)	10 (52.6%)	<0.001
ED room (*n*, %)	501 (26.3%)	417 (27.4%)	53 (18.4%)	23 (29.9%)	8 (42.1%)	
Other hospitals	299 (15.7%)	244 (16.0%)	30 (10.4%)	24 (31.2%)	1 (5.26%)	
General practitioner	24 (1.26%)	21 (1.38%)	1 (0.35%)	2 (2.60%)	0 (0.00%)	

ED: emergency department, BP: blood pressure, PACI: partial anterior cerebral circulation infarction, TACI: total anterior cerebral circulation infarction, POCI: posterior cerebral infarction, LACI: lacunar cerebral infarction, SC: stroke code, EMS: emergency medical system.

Vascular risk factors and comorbidities were similar among all patients regardless of stroke subtype, except for patients with stroke mimics who were younger and had fewer risk factors and comorbidities compared to patients with ischemic stroke, ICH, and TIA. Patients with ICH had higher stroke severity and higher systolic BP than patients with other subtypes. Regarding the origin of the stroke code alert, EMS was the most frequent source of activation in all stroke code subtypes, especially in ICH patients. The median time from symptom onset to arrival at the emergency room was 170 min for all patients and was 33 min shorter for ICH than ischemic stroke patients (146 [76–385] min versus 179 [80–553] min; *p* = 0.026).

**Figure 1:**
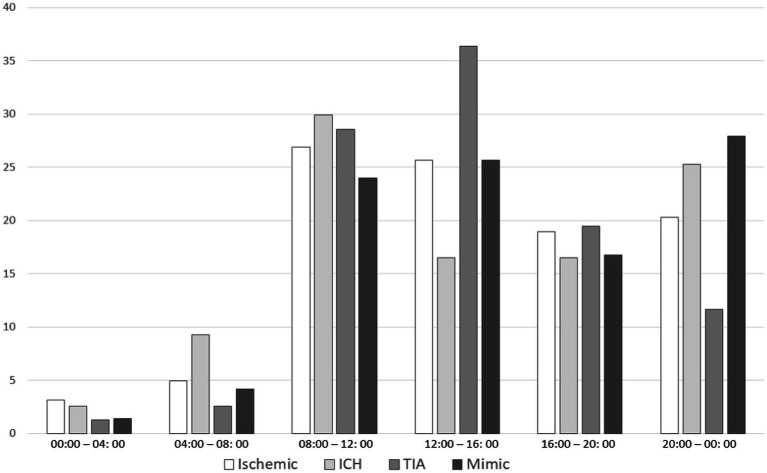
Distribution of stroke subtypes by the onset of symptoms. Figure 1 shows the percentage of stroke subtypes (ischemic, intracerebral hemorrhage (ICH), TIA, and mimic) by 4-h periods of day. Each graph bar value corresponds to the proportion of patients in each period within their respective stroke subtype group. Wake-up strokes have been excluded from this analysis.

**Figure 2:**
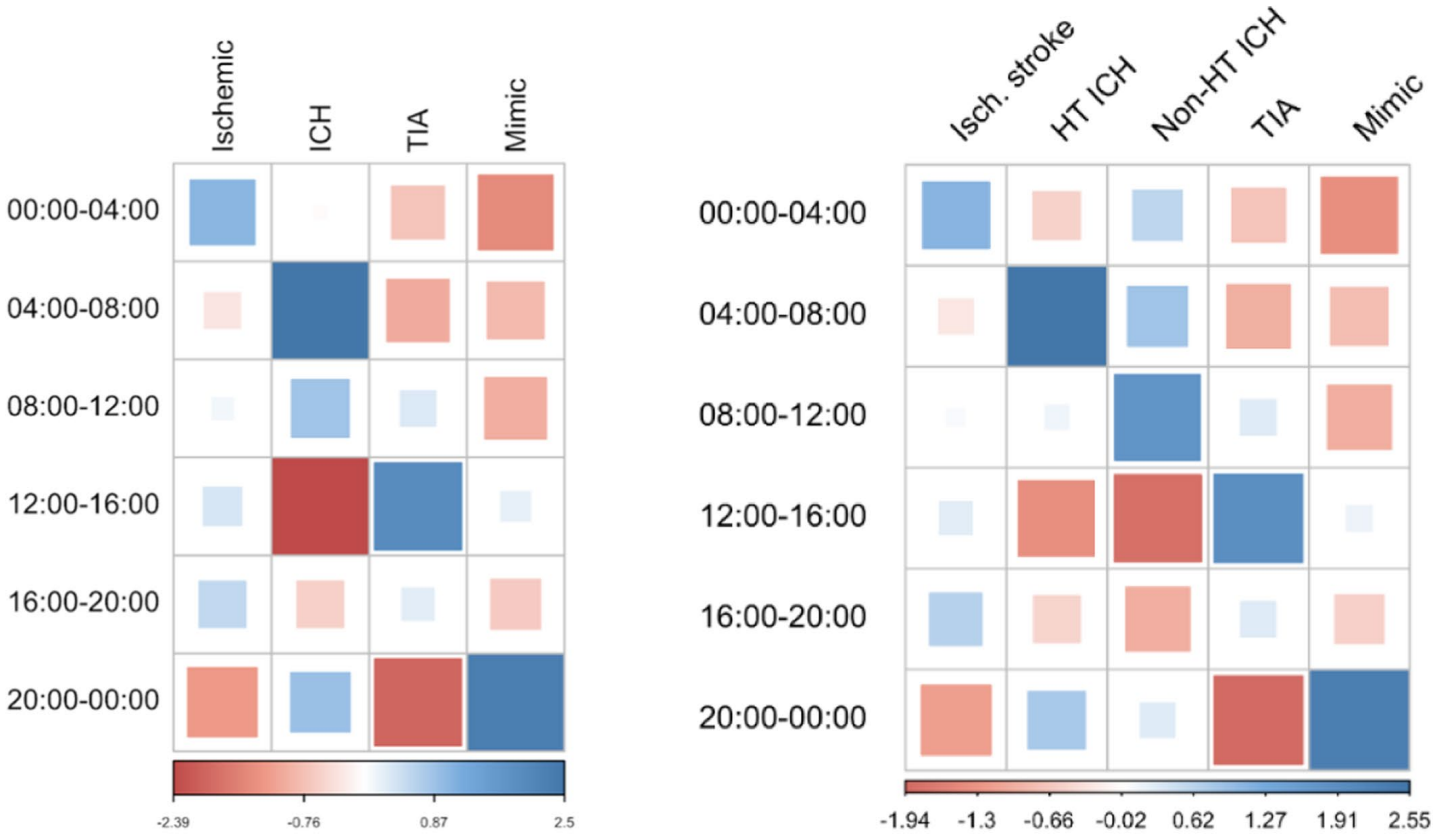
Matrix showing excess cases in each time period according to the stroke subtype. Figure 2 compares the number of patients of each stroke subtype in their respective time slot and the theoretically expected number of cases, in relation to the observed cases of other subtypes. Blue is associated with a positive correlation (there are more cases than expected for the subtype and hour of day), and red is associated with a negative distribution (there are fewer cases than expected). ICH is positively correlated in the 04–08 h slot and negatively correlated in the 12–16 h slot. TIA is positively correlated in the 12–16 h slot while mimic in the 20–00 h slot. Ischemic stroke is more equally distributed throughout the day. The second image shows the correlation between ICH strokes by their etiology, divided by hypertensive, and non-hypertensive etiology, and the theoretical number of cases that should appear in a normal distribution for each group. Hypertensive ICH shows a preference in the 04–08, 12–16, 16–20, and 20–00 h time slots. ICH: intracerebral hemorrhage; TIA: transient ischemic attack.

Wake-up or uncertain onset stroke patients represent 30.3% of the sample (36.6% of ischemic strokes, 35.3% of ICH, 13.5% of TIA, and 3.2% of mimic). Regarding the circadian rhythm of the time of symptoms onset, after excluding wake-up or uncertain onset stroke, most of the patients were distributed in the 8–12 and 12–16 h period, regardless of stroke subtype. The symptom onset time distribution according to stroke subtypes is shown in Table 2 and Figure 1. Statistically significant differences were found between time distributions depending on stroke subtype, showing an excess of cases than expected of ICH in the 4–8 h period (dawn), TIA in the 12–16 h period (afternoon), and stroke mimic in the 20 h period (evening). A negative association was found for ICH in the 12–16 h (afternoon) period and TIA in the 20 h period (evening) (Figure 2). Importantly, when we analyzed the subgroup of 300 patients with ICH according to their etiology (165 hypertensive, 87 no hypertensive, and 48 with unknown etiology), we observed that ICH secondary to hypertension had a higher frequency of appearance in the 4–8 h period than other ICH etiologies (Figure 2).

**Table 2:** Number of patients distributed by etiology and the time of onset of symptoms.

*N* (%)	Ischemic stroke *N* = 1,008	ICH *N* = 194	TIA *N* = 77	Mimic *N* = 358
0–4	32 (3.17)	5 (2.58)	1 (1.30)	5 (1.40)
4–8	50 (4.96)	18 (9.28)	2 (2.60)	15 (4.19)
8–12	271 (26.88)	58 (29.90)	22 (28.57)	86 (24.02)
12–16	259 (25.69)	32 (16.49)	28 (36.36)	92 (25.70)
16–20	191 (18.95)	32 (16.49)	15 (19.48)	60 (16.76)
20–00	205 (20.34)	49 (25.26)	9 (11.69)	100 (27.93)

Percentage (%) refers to the proportion of patients within their respective stroke subtype group (column). Wake-up and uncertain onset stroke patients are excluded from this analysis. ICH: intracerebral hemorrhage; TIA: transient ischemic attack.

**Figure 3:**
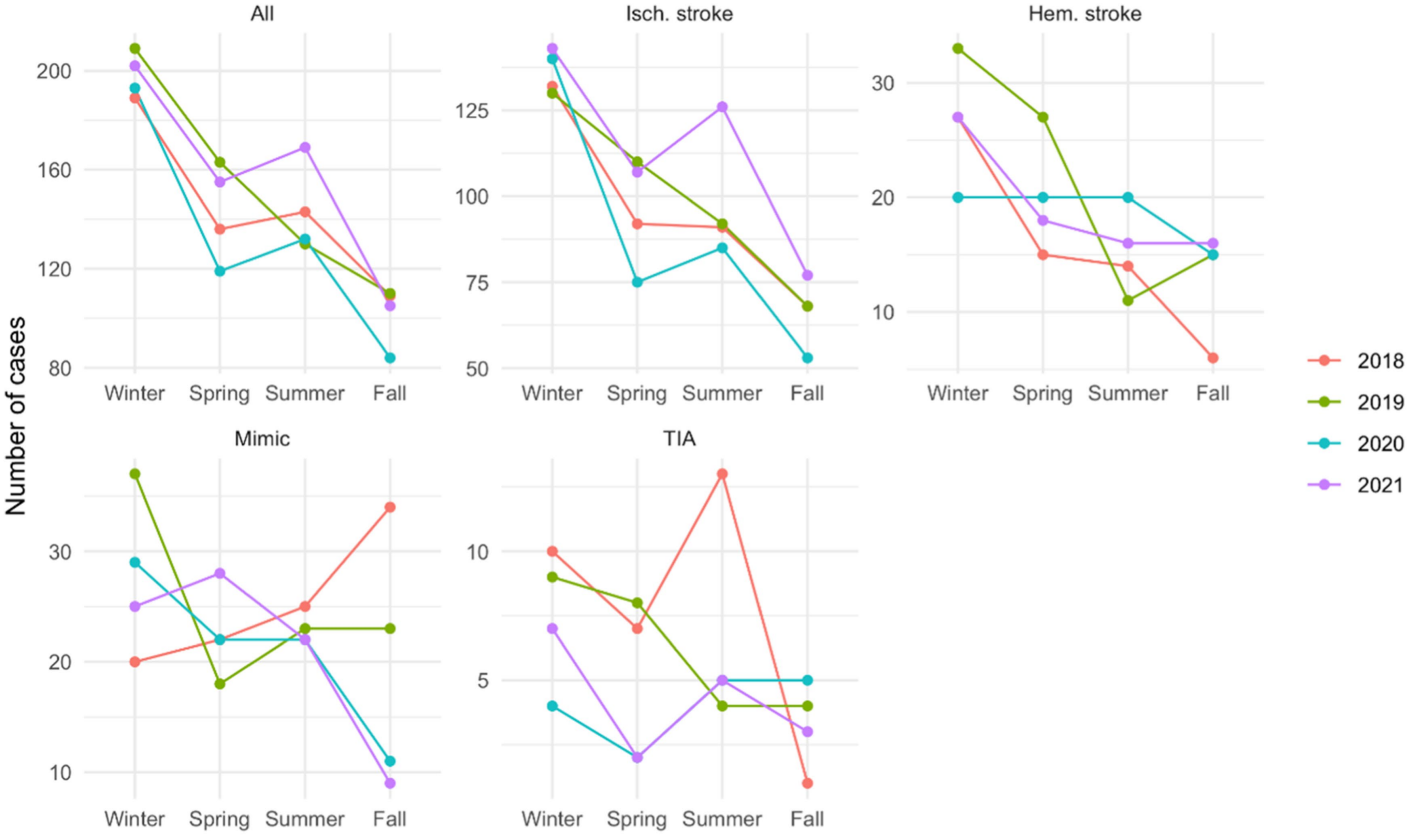
Stroke distribution by seasons, in the total cohort, and by the stroke subtype Number of cases by each season, in the total cohort and by stroke subtype, showing a higher peak in winter for ischemic (*p* < 0.01) and hemorrhagic stroke (*p* = 0.05), while the others did not show a specific pattern.

A seasonality pattern was observed when all the cases were studied together, with a higher peak in winter (*p* = 0.01). When analyzed by stroke subtype, ischemic (*p* < 0.01) and hemorrhagic strokes (*p* = 0.05) showed the same seasonality pattern, while the others did not show a specific pattern (Figure 3).

**Figure 4:**
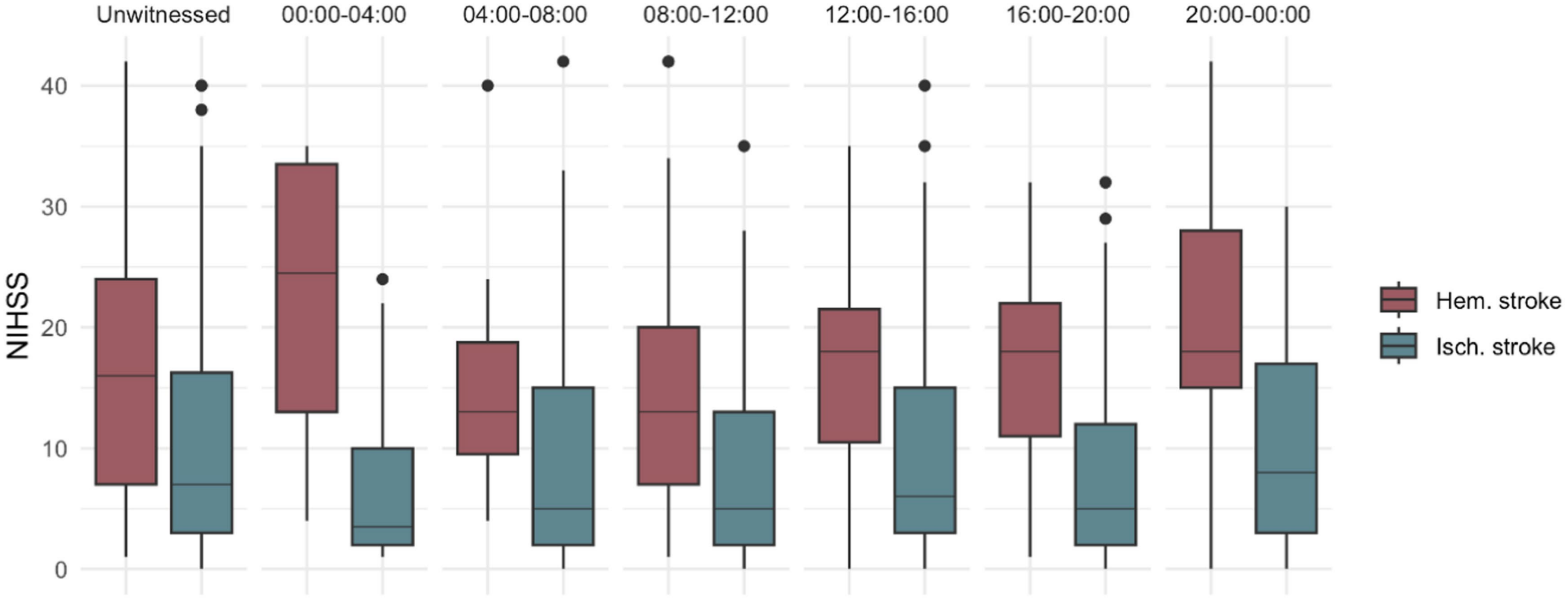
Ischemic and hemorrhagic stroke differences by severity along the day NIHSS comparison between ischemic and ICH, showing higher NIHSS for ICH in all-time distributions, a higher severity between 20–00 for ischemic stroke patients and between 00–04 for ICH patients.

Stroke severity was significantly higher for ICH than other stroke subtypes across all periods of the day, including unwitnessed stroke (*p* < 0.001). For patients with ischemic stroke, a higher severity was observed for patients with onset time between 20:00 and 00:00 h (*p* = 0.014), while ICH patients showed a non-significant trend to have more severe symptoms between 00:00 and 4:00 h (*p* = 0.07) compared with other day periods (Figure 4).

## Discussion

Whether there is a specific circadian rhythm profile for onset symptoms according to stroke code subtype remains unresolved. The present study shows in a large sample of stroke code patients an association between symptoms onset and stroke subtype: a predominance of ICH at dawn, TIA in the afternoon, and stroke mimic in the evening, whereas ischemic stroke is more evenly distributed through the different periods of the day.

Our findings are in accordance with previous studies that also showed a temporal distribution of stroke onset throughout the day, showing a double-peak 24-h pattern with a higher incidence of symptoms onset in the morning and afternoon (7–9). However, we observed a greater number of ICH cases than other subtypes in the dawn period. Saver et al. found similar results in an analysis of the FAST-MAG patients, with a high ratio of ICH/ischemic stroke in the early morning (32). The current study reinforces that the proportion of ischemic/ICH subtypes shows a predominance of ICH in this early morning period compared to other periods of the day. Importantly, this is the first time that mimic strokes have been studied, showing a predominance in the afternoon.

The distribution of onset time in ICH and ischemic stroke may probably reflect changes in circadian rhythm, paralleling changes in BP that peak in the morning (33). This hypothesis is supported by our results showing that the hypertensive etiology of ICH was the most common etiology of ICH at dawn, and both ischemic and ICH were highest in the morning. Furthermore, seasonal changes in BP throughout the year could also explain, in part, the highest incidence of stroke in winter (34).

On the other hand, it is well known that the severity of stroke is strongly correlated with the subtype. As a novelty, our study analyzes the differences in the severity of the symptoms throughout the day, according to the stroke code subtype. We observed a different distribution within each stroke subtype, with a peak of higher severity at dawn for ICH and in the evening for ischemic stroke. These findings align with previous studies in the literature (35). The more severe symptoms in ICH in the early hours of the day (00:00–08:00 h) might be the basis for stricter BP control at night. Further studies might be performed to discern those patients with a worse systolic BP pattern at night to individualize treatment.

Regarding patients with stroke mimics, we observed that they show a very different time pattern of symptom presentation compared to stroke patients, as they are unlikely to start on awakening or unwitnessed, being more frequent in the evening. This fact, together with the low frequency of vascular risk factors and comorbidities in these patients, distinguishes them as a clearly differentiated group from the other types of strokes.

These findings open up a question about the underlying pathophysiological mechanisms and triggers for each stroke subtype. Moreover, they offer relevant data for the development of technological tools that take into account clinical and epidemiological factors with the aim of differentiating between ischemic strokes, ICH, or stroke mimics. Knowing the specific time distribution of stroke subtypes might improve stroke protocols and pre-hospital strategies, helping to classify patients in the emergency room and aiding the clinician in decision-making. This study opens several lines of research, encouraging further investigation into the subcategories and etiology of stroke. The pathophysiological conditions, topography, clinical characteristics, and prognosis vary between lacunar, cardioembolic, and atherosclerotic strokes (36). It is known that the cardioembolic cause is a more frequent etiology in ischemic strokes in the territory of the anterior cerebral artery (37), so imaging studies with emphasis on the location of the lesion can be included in subsequent studies.

This study has some limitations: The current analysis does not study differences between the different etiologies of ischemic stroke (lacunar, cardioembolic, and atherothrombotic) or according to the presence of large vessel occlusion. Regarding stroke mimic, we have no detailed data about the final diagnosis of stroke mimic (epileptic seizure, functional neurological disorder, and hypoglycemia, among others). It is a single-center study, which may have inherent potential for bias in patient selection and make it difficult to extrapolate findings to broader populations. The present study, being descriptive, could only describe hemodynamic characteristics recorded at admission. However, the lack of physiological data at the onset of symptoms of stroke during the pre-hospital period makes it difficult to draw specific data on circadian rhythm for a better explanation of the behavior of this disease. Finally, regarding the differences related to seasonality, it must be taken into account that our study includes a single geographical area that could be exposed to social and demographic factors, which can affect the number of cases according to the month of the year. As a strength of the study, we highlight that it is a large cohort of patients with prospectively collected data, which ensures the quality of their collection.

In conclusion, the data presented in this study provide a comprehensive picture of stroke onset time patterns, showing a similar distribution of ischemic and ICH, with a slight peak of ICH at dawn and stroke mimic in the evening. Further understanding of stroke subtypes may lead to better emergency code management by the emergency room staff and the patients themselves.

## Data availability statement

The original contributions presented in the study are included in the article/supplementary material, further inquiries can be directed to the corresponding author.

## Ethics statement

The studies involving humans were approved by the Ethical Committee at Germans Trias i Pujol University Hospital approved the study (registry code PI-19-080). The studies were conducted in accordance with the local legislation and institutional requirements. Written informed consent for participation was not required from the participants or the participants’ legal guardians/next of kin in accordance with the national legislation and institutional requirements. Written informed consent was obtained from the individual(s) for the publication of any potentially identifiable images or data included in this article.

## Author contributions

AM: Conceptualization, Data curation, Formal analysis, Investigation, Methodology, Validation, Writing – original draft, Writing – review & editing. AV: Conceptualization, Data curation, Formal analysis, Software, Writing – review & editing. NR: Writing – review & editing, Data curation, Investigation, Methodology. BY: Writing – review & editing, Data curation, Investigation, Methodology. JC: Writing – review & editing, Data curation, Investigation, Methodology. BF-P: Writing – review & editing, Data curation, Investigation, Methodology. M-CL: Writing – review & editing, Data curation, Investigation, Methodology. MSM: Writing – review & editing, Data curation, Investigation, Methodology. MH-P: Writing – review & editing, Data curation, Investigation, Methodology. AB: Writing – review & editing, Data curation, Investigation, Methodology. LD: Writing – review & editing, Data curation, Investigation, Methodology. MG: Writing – review & editing, Data curation, Investigation, Methodology. MMT: Writing – review & editing, Conceptualization, Formal analysis, Project administration, Resources, Supervision, Validation, Visualization. NP: Validation, Visualization, Writing – original draft, Conceptualization, Data curation, Formal analysis, Funding acquisition, Investigation, Methodology, Project administration, Resources, Supervision.
